# Gastric mucin phenotype indicates aggressive biological behaviour in early differentiated gastric adenocarcinomas following endoscopic treatment

**DOI:** 10.1186/s13000-021-01122-2

**Published:** 2021-07-13

**Authors:** Kai Song, Qi Yang, Yu Yan, Xiaoyan Yu, Kanlun Xu, Jinghong Xu

**Affiliations:** Department of Pathology, The Second Affiliated Hospital, Zhejiang University School of Medicine, Hangzhou, China

**Keywords:** Gastric cancer, Early stage, Mucin core protein, Gastric Mucin phenotype, Biological behaviour

## Abstract

**Background:**

The distribution of mucin phenotypes and their relationship with clinicopathological features in early differentiated gastric adenocarcinomas in a Chinese cohort are unknown. We aimed to investigate mucin phenotypes and analyse the relationship between mucin phenotypes and clinicopathological features, especially biological behaviours, in early differentiated gastric adenocarcinomas from endoscopic specimens in a Chinese cohort.

**Methods:**

Immunohistochemical staining of CD10, MUC2, MUC5AC, and MUC6 was performed in 257 tissue samples from patients with early differentiated gastric adenocarcinomas. The tumour location, gross type, tumour size, histological type, depth of invasion, lymphovascular invasion, mucosal background and other clinicopathological parameters were evaluated. The relationship between mucin phenotypes and clinicopathological features was analysed with the chi-square test.

**Results:**

The incidences of gastric, gastrointestinal, intestinal and null phenotypes were 21 %, 56 %, 20 and 3 %, respectively. The mucin phenotypes were related to histology classification (*P <* 0.05). The proportion of the gastric phenotype became greater during the transition from differentiated to undifferentiated (*P <* 0.05). Complete intestinal metaplasia was higher in the gastric and intestinal phenotypes than in the gastrointestinal phenotype (*P <* 0.05). Tumours with poorly differentiated adenocarcinoma were mainly of the gastric phenotype, which was significantly higher than that of purely differentiated tubular adenocarcinoma (*P* < 0.05), and the depth of invasion in the mixed type was deeper (*P* < 0.05). Neither recurrence nor metastasis was detected.

**Conclusions:**

The mucin phenotype of early-differentiated gastric adenocarcinoma has clinical implications, and the gastric phenotype has aggressive biological behaviour in early differentiated gastric cancers, especially in those with poorly differentiated adenocarcinoma or papillary adenocarcinoma components.

## Background

Gastric cancer (GC), one of the most common human cancers worldwide, is a disease with multiple pathogenic factors, various prognoses and different responses to treatments. Thus, properly distinguishing those with worse prognoses from those with better prognoses appears to be significantly important. Four different morphology -based classification systems exist, the World Health Organization (WHO/2019) [1], the Japanese Gastric Cancer Association (JGCA/2017) [1], Laurén [2] and Nakamura [3]. According to the WHO classification, GCs are subclassified into papillary, tubular, poorly cohesive, mucinous and mixed types. In the JGCA classification, the subtypes are papillary (pap), tubular (tub), poorly differentiated (por), signet-ring cell (sig), and mucinous (muc), which are similar to the subtypes used by the WHO. GCs are divided into intestinal and diffuse types using Laurén’s classification or into differentiated and undifferentiated types based on Nakamura’s classification [2–4]. The differentiated type contains pap, tub1, and tub2 according to the JGCA classification and papillary and well/moderately differentiated adenocarcinoma according to the WHO classification. These different histological types exhibit distinct biological behaviours.

The mucous produced by cancers is one of the factors determining the nature of biological behaviour. The main component of mucous is a high-molecular-weight glycoprotein called mucin [5]. As cancer progresses, the nature of the mucous changes relative to the degree of biological malignancy. In the 1990 s, with the progress of structural analysis of mucin and the widespread use of monoclonal antibodies to the core protein of mucin, a mucin phenotype subclassification emerged. Mucin phenotype subclassification was entirely based on the mucin expression pattern, independent of histological features. Thus, GCs are classified into gastric, intestinal, gastrointestinal and null mucin phenotypes [4–6]. Previous studies have reported that the gastric phenotype has a higher potential for invasion and metastasis than the intestinal type, which results in a worse prognosis of GCs [7–11]. However, studies have mostly focused on advanced gastric cancers, and early gastric cancers are rarely investigated.

Early gastric cancer (EGC) is defined as tumour invasion confined to the mucosa and submucosa, irrespective of regional lymph node metastasis [12]. Endoscopic mucosal resection (EMR) and endoscopic submucosal dissection (ESD) are used as treatments for some intramucosal carcinomas and submucosal lesions, which have a very low probability of lymph node metastasis [13, 14].

To our knowledge, there is no research exploring biological role of mucin phenotypes in EGCs using EMR/ESD by Chinese investigators. Little information is available on the effects of mucin phenotypes on the clinicopathological features of EGCs in a Chinese cohort. Accordingly, we examined mucin expression and mucin phenotypes and explored mucin phenotype clinicopathological characteristics and biological behaviour.

## Methods

### Patients and tissue specimens

Our study consisted of 257 consecutive patients who underwent EMR/ESD for differentiated EGCs between January 2012 and June 2018 at The Second Affiliated Hospital of Zhejiang University, China. The group comprised 182 men and 75 women with an age range of 29 to 87 (mean 64) years old.

The location of each lesion was classified in terms of the upper (27 cases), middle (46 cases) and lower (184 cases) thirds of the stomach. The size of each lesion was measured by the maximum diameter, which ranged from 0.1 to 6.5 (mean 1.5) centimetres. The protruding category (52 cases) included type 0-I and 0-IIa, the depressed (102 cases) category contained type 0-IIc and III, and all the other cases were considered under the protruding and depressed category (103 cases). Based on the WHO and JGCA classification, the EGCs were subclassified into well differentiated tubular adenocarcinoma (well-diff/tub 1, 198 cases), moderately differentiated tubular adenocarcinoma (mod-diff/tub 2, 37 cases), papillary adenocarcinoma (3 cases) and mixed (tub-pap/sig/por, 19 cases). In the mixed cases, the undifferentiated components (sig/por) were less than 50 %.

### Immunohistochemistry

All specimens were fixed with 10 % buffered formalin, embedded in paraffin, cut into 4-µm-thick sections, and subjected to haematoxylin and eosin (HE) staining. MUC2 was detected by mAb Ccp58 (Zsbio, 1:100), MUC5AC by mAb MRQ-19 (Zsbio, 1:100), MUC6 by mAb MRQ-20 (Zsbio, 1:100), and CD10 (Zsbio, 1:100) by immunohistochemistry (IHC). IHC was performed by using the Ventana NexES Staining System (Roche, Benchmark®XT). The marker CD10 exhibited both cytoplasmic and glandular luminal reactivity, whereas MUC5AC, MUC6 and MUC2 exhibited only cytoplasmic reactivity. The staining results were categorized as positive when at least one single cell among the carcinoma cells was stained and negative when none of the carcinoma cells were stained [6].

### Classification of mucin phenotype

Based on MUC5AC, MUC6, MUC2 and CD10, EGCs can be classified into the gastric phenotype (G-type), gastrointestinal phenotype (GI-type), intestinal phenotype (I-type) and null phenotype (N-type) [4, 6, 15–17]. The following criteria were used for the classification of mucin phenotypes: ① the G-type shows positive staining for at least one of the MUC5AC and MUC6, while CD10 and MUC2 are both negative; ② the I-type shows positive staining for CD10 and/or MUC2, while MUC5AC and MUC6 are both negative; ③ the GI-type shows positive staining for marker CD10 and/or MUC2 associated with one of the markers MUC5AC and MUC6 positive; and④ if none of the four markers are positive, the phenotype is classified as N-type.

In addition, among the GI-types, we labelled one expressing more MUC5AC and/or MUC6 than MUC2 and CD10 as a gastric-predominant GI phenotype (GI-G type); otherwise, we labelled it as an intestinal-predominant phenotype (GI-I type).

### Statistical analysis

Associations between mucin expression profiles and clinicopathological parameters were examined by the chi-square test or Fisher’s exact test. Statistical significance was established to be *P* < 0.05. Statistical calculations were performed with IBM SPSS Statistics (version 23.0).

## Results

### Expression of mucin markers and mucin phenotype in early gastric cancers

**Fig. 1:**
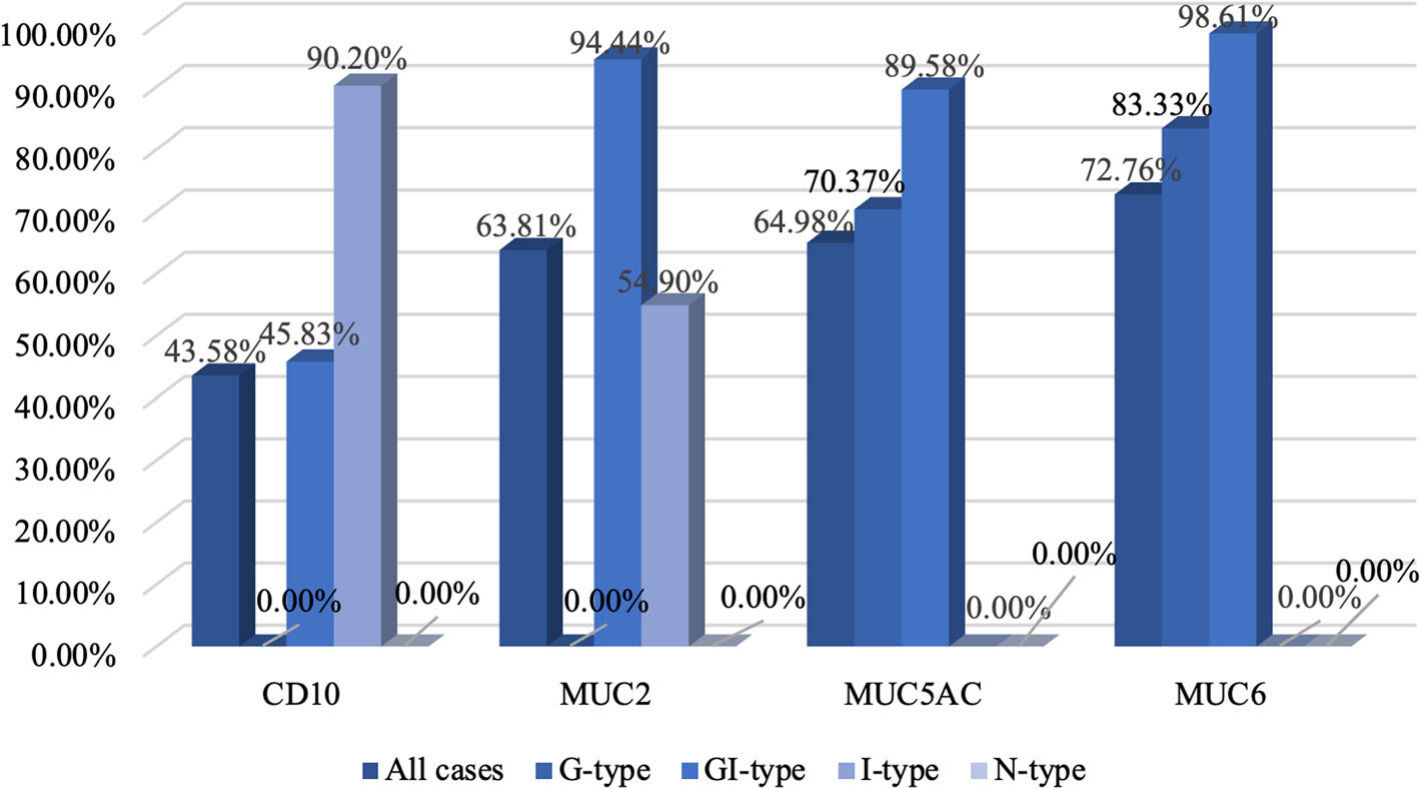
The proportion of CD10, MUC2, MUC5AC, and MUC6 in each mucin phenotype

The expression percentages of CD10, MUC2, MUC5AC and MUC6 in all EGCs were 43.58 % (112/257), 63.81 % (164/257), 64.98 % (167/257) and 72.76 % (187/257) respectively (Fig. 1).

**Fig. 2:**
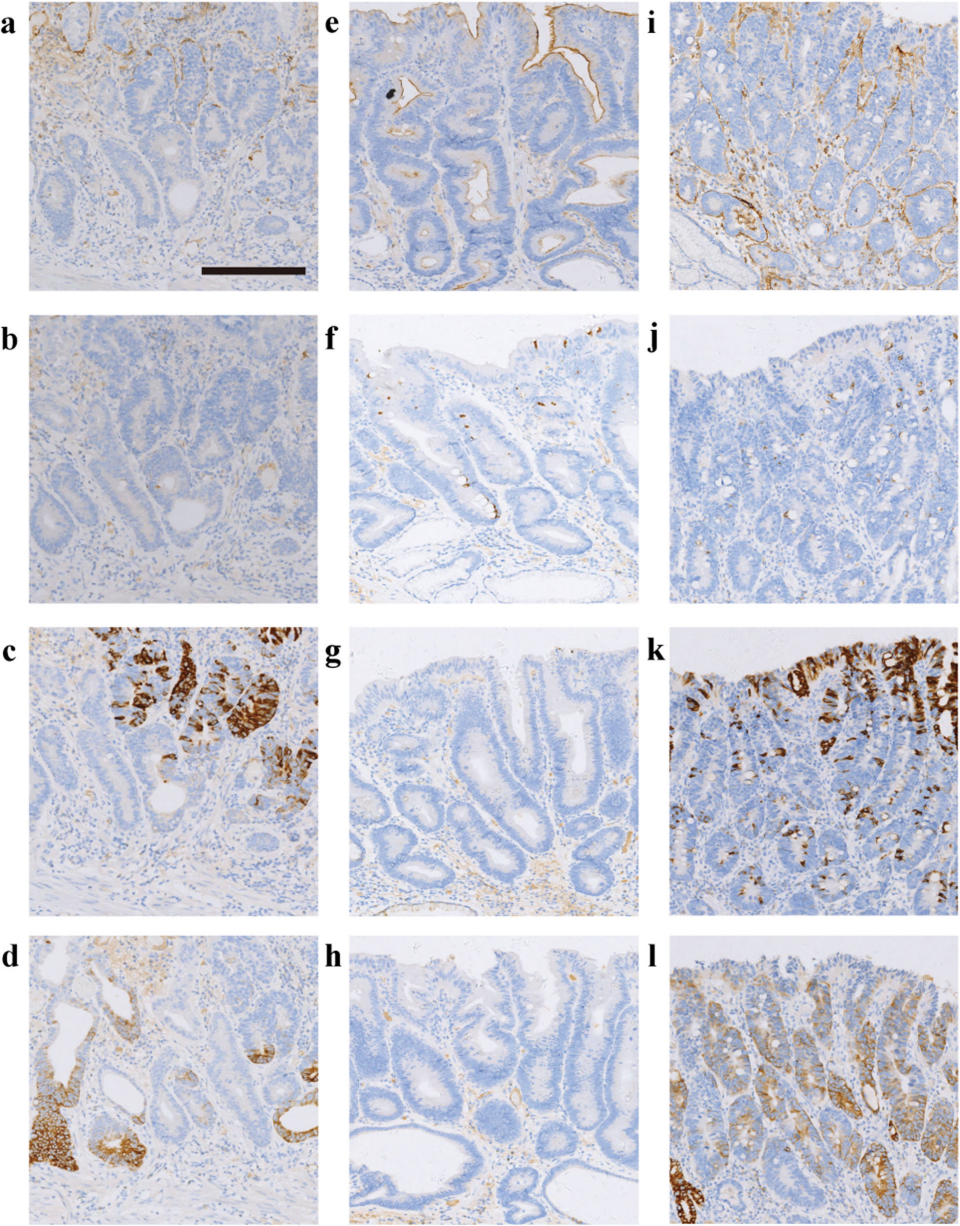
IHC staining of the gastric phenotype, intestinal phenotype and gastrointestinal phenotype. G-type (**a-d**) shows negative staining for CD10 (**a**) and MUC2 (**b**) and positive staining for MUC5AC (**c**) and MUC6 (**d**). In contrast, I-type (**e-h**) shows CD10 (**e**) and MUC2 (**f**) marker positivity and negative staining for MUC5AC (**g**) and MUC6 (**h**) negative. GI-type (**i-l**) shows positive staining for CD10 (**i**), MUC2 (**j**),MUC5AC (**k**) and MUC6 (**l**). (Scale bar =200 μm, HE and IHC ×10)

Two hundred fifty-seven EGCs were classified as G-type (21 %, 54/257), GI-type (56 %, 144/257), I-type (20 %, 51/257) and N-type (3 %, 8/257). The GI-type contained the GI-G type (72 %, 103/144) and GI-I type (28 %, 41/144). There were more cases of G-type and GI-G type (61 %, 157/257) than I-type and GI-I type (36 %, 95/257) (Fig. 2).

### Relationship between mucin phenotype and clinicopathological features

The relationship between mucin phenotype and clinicopathological features is summarized in Table 1. The mucin phenotypes were significantly related to the JGCA and WHO classifications (*P <* 0.05), but the parameters of sex, age, margin, colour, tumour size, gross type, depth of invasion, and lymphovascular invasion did not significantly differ among those mucin phenotypes (*P >* 0.05). The I-type had the highest proportion of differentiated EGCs among the four mucin phenotypes (100.0 % vs. 79.7 % vs. 83.1 % vs. 87.5 %, *P* = 0.027). The G-type group had a higher proportion of tub-por/sig and pap/tub-pap cases than the I-, GI- and N-type groups (20.4 % vs. 7.0 % vs. 0.0 % vs. 12.5 %, *P* = 0.027) according to the JGCA classification. According to the WHO classification, the G-type had more mixed histological components (18.5 % vs. 5.6 % vs. 0.0 % vs. 12.5 %, *P* = 0.006) than the other mucin phenotypes.

**Table 1:** Relationship between Mucin Phenotypes and Clinicopathological Features

	G-type		GI-type		I-type	N-type	χ2 value	*P value*
**Sex**							0.671	0.906
Male	40(74.1 %)		99(68.8 %)		37(72.5 %)	6(75.0 %)		
Female	14(25.9 %)		45(31.2 %)		14(27.5 %)	2(25.0 %)		
**Age**							0.412	0.945
≤ 64 years	28(51.9 %)		75(52.1 %)		29(56.9 %)	4(50.0 %)		
>64 years	26(48.1 %)		69(47.9 %)		22(43.1 %)	4(50.0 %)		
**Tumor Size**							2.356	0.502
≤ 2 cm	34(63.0 %)		99(68.8 %)		32(62.7 %)	7(87.5 %)		
>2 cm	20(37.0 %)		45(31.2 %)		19(37.3 %)	1(12.5 %)		
**Margin**							4.269	0.224
Distinct	35(64.8 %)		111(77.1 %)		40(78.4 %)	5(62.5 %)		
Indistinct	19(35.2 %)		33(22.9 %)		11(21.6 %)	3(37.5 %)		
**Color**							3.423	0.337
Darken	31(57.4 %)		75(52.1 %)		34(66.7 %)	5(62.5 %)		
Faded	23(42.6 %)		69(47.9 %)		17(33.3 %)	3(37.5 %)		
**Tumor Location**							5.819	0.407
Upper	4(7.4 %)		15(10.4 %)		8(15.7 %)	0(0.0 %)		
Middle	12(22.2 %)		21(14.6 %)		12(23.5 %)	1(12.5 %)		
Low	38(70.4 %)		108(75.0 %)		31(60.8 %)	7(87.5 %)		
**Tumor Location**							0.787	0.842
EGJ	4(7.4 %)		7(4.9 %)		3(5.9 %)	0(0.0 %)		
No-EGJ	50(92.6 %)		137(95.1 %)		48(94.1 %)	8(100.0 %)		
**Gross type**							8.943	0.162
Protruding	10(18.5 %)		32(22.2 %)		10(19.6 %)	0(0.0 %)		
Depressed	16(29.6 %)		57(39.6 %)		26(51.0 %)	3(37.5 %)		
Protruding-Depressed	28(51.9 %)		55(38.2 %)		15(29.4 %)	5(62.5 %)		
**JGCA Classification**							16.870	0.027
tub1	34(62.9 %)		112(77.8 %)		45(88.2 %)	7(87.5 %)		
tub2	9(16.7 %)		22(15.2 %)		6(11.8 %)	0(0.0 %)		
tub-por/sig	7(13.0 %)		7(4.9 %)		0(0.0 %)	1(12.5 %)		
pap/tub-pap	4(7.4 %)		3(2.1 %)		0(0.0 %)	0(0.0 %)		
**WHO Classification**							18.052	0.017
Tubular, well-diff	34(62.9 %)		112(77.8 %)		45(88.2 %)	7(87.5 %)		
Tubular, mod-diff	9(16.7 %)		22(15.2 %)		6(11.8 %)	0(0.0 %)		
Papillary	1(1.9 %)		2(1.4 %)		0(0.0 %)	0(0.0 %)		
Mixed	10(18.5 %)		8(7.0 %)		0(0.0 %)	1(12.5 %)		
**Depth of Invasion**							1.360	0.681
M	47(87.0 %)		132(91.7 %)		46(90.2 %)	8(100.0 %)		
SM	7(13.0 %)		12(8.3 %)		5(9.8 %)	0(0.0 %)		
**Lymphovascular invasion**							2.541	0.687
(+)	1(1.9 %)		1(0.7 %)		0(0.0 %)	0(0.0 %)		
(-)	53(98.1 %)		143(99.3 %)		51(100.0 %)	8(100.0 %)		

+, present; - absent

*EGJ* esophagogastric junction, *well-diff* well differentiated, *mod-diff* moderately differentiated, *M* mucosa, *SM* submucosa

### Relationship between mucin phenotypes and background mucosa

Intestinal metaplasia (IM) of background mucosa was observed in 199 of 249 (79.9 %) cases (G-, GI- and GI-type), including 38 cases of incomplete IM and 161 cases of complete IM. IM did not significantly differ among mucin phenotypes (*P >* 0.05). However, the presence of incomplete and complete IM was significantly different in distinct mucin phenotypes (*P* = 0.004, *P* = 0.018). The presence of incomplete IM in GI-type EGCs was higher than that in the G-type and I-type EGCs (21.5 % vs. 9.3 % vs. 3.9 %). In the contrast, 77.8 % (42/54) of G-type EGCs and 70.6 % (36/51) of I-type EGCs exhibited complete IM, which was higher than the 57.6 % (83/114) of GI-type EGCs. The IM status of the background mucosa and the relationship with mucin phenotypes are shown in Table 2.

**Table 2:** Relationship between mucin phenotypes and background mucosa

	G-type	GI-type	I-type	χ2 value	*P* value
Intestinal metaplasia				2.685	0.265
(+)	47(87.1 %)	114(79.2 %)	38(74.5 %)		
(-)	7(12.9 %)	30(20.8 %)	13(25.5 %)		
Incomplete intestinal metaplasia				10.948	0.004
(+)	5(9.3 %)	31(21.5 %)	2(3.9 %)		
(-)	49(90.7 %)	113(78.5 %)	49(96.1 %)		
Complete intestinal metaplasia				7.957	0.018
(+)	42(77.8 %)	83(57.6 %)	36(70.6 %)		
(-)	12(22.2 %)	61(42.4 %)	15(29.4 %)		

+, present; - absent

### Biological behaviour of mucin phenotypes

**Fig. 3:**
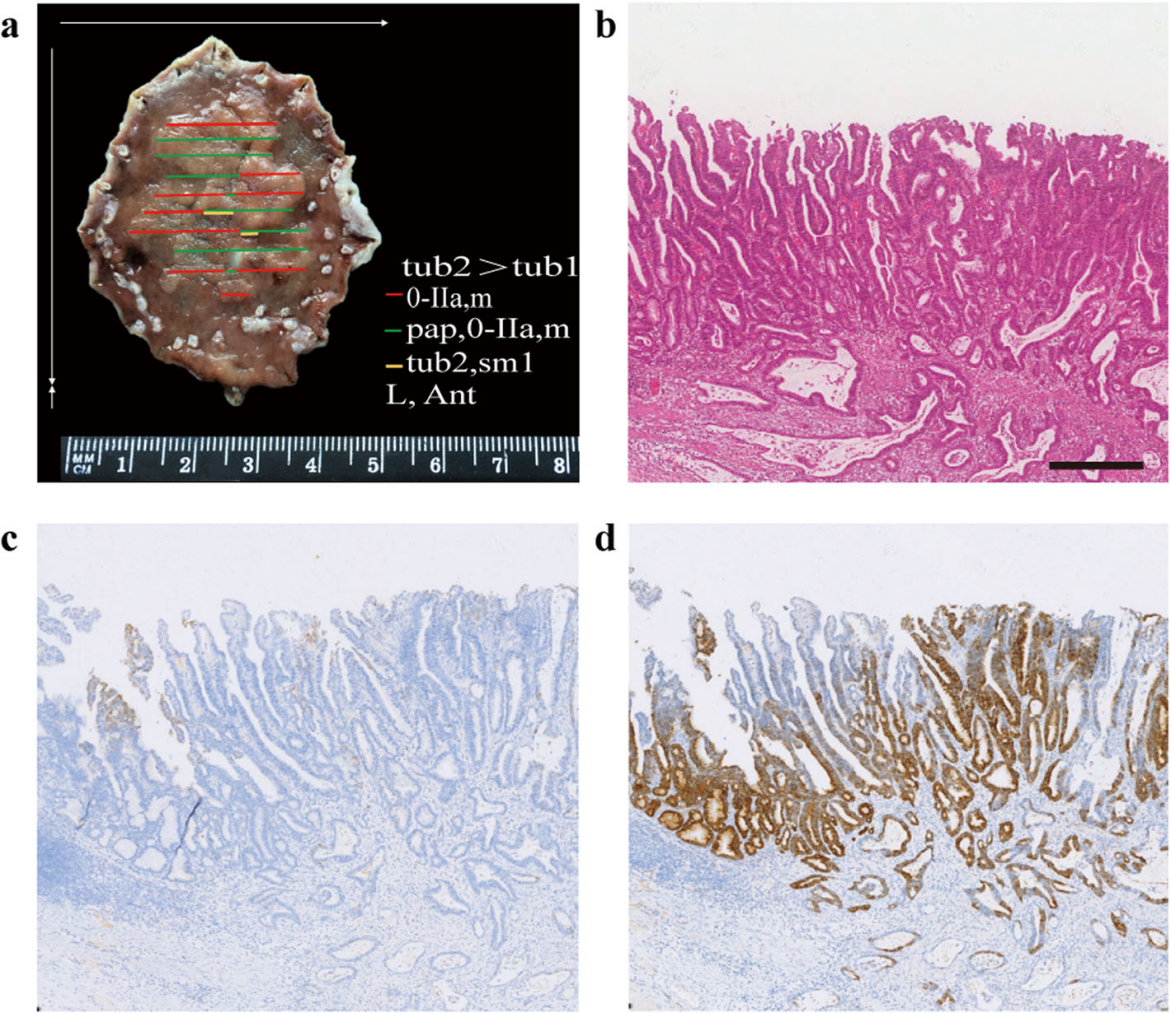
Tubular adenocarcinoma mixed with papillary adenocarcinoma showing the gastric mucin phenotype. Reconstructive map (**a**). H&E staining showing papillary structure on the surface and tubular adenocarcinoma in the submucosa; invasive depth is 300 μm (**b**). Focal positive staining for MUC5AC (**c**) and strongly positive staining for MUC6 (**d**), invasive tubular adenocarcinoma showing MUC5AC negative (**c**) and MUC6 positive (**d**) status. (Scale bar =400 μm, HE and IHC ×10)

**Fig. 4:**
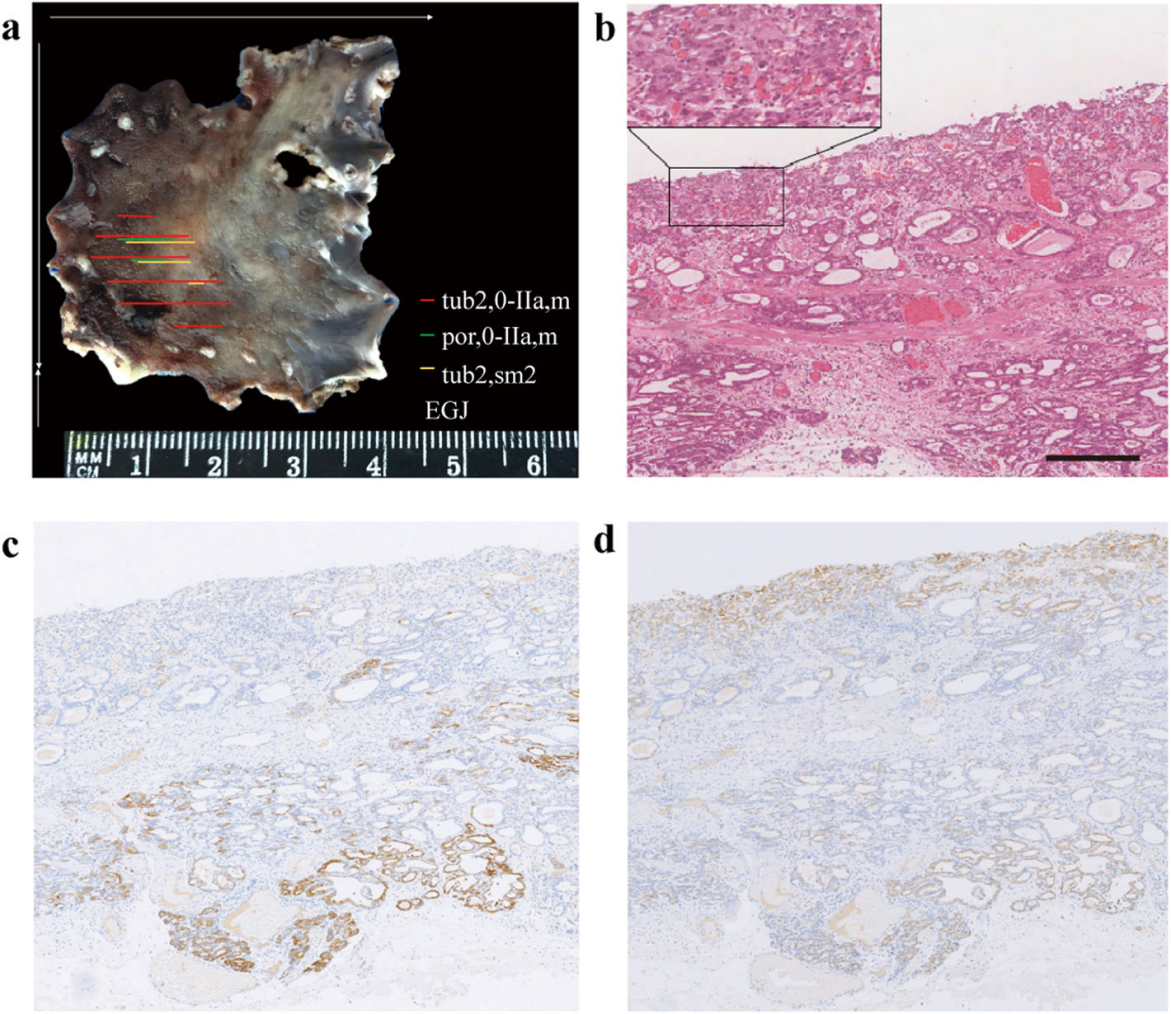
Tubular adenocarcinoma mixed with poorly differentiated carcinoma showing the gastric mucin phenotype. Reconstructive map (**a**). H&E staining showing poorly differentiated carcinoma in the mucosa and tubular adenocarcinoma in the submucosa; the invasive depth is 2500 μm (**b**). Positive staining for MUC5AC (**c**) and MUC6 (**d**), and invasive tubular adenocarcinoma showing MUC5AC (**c**) and MUC6 (**d**) positivity. (Scale bar =400 μm, HE and IHC x10)

In addition to tubular adenocarcinoma components, 22 of the 257 cases also contained components of papillary adenocarcinoma, poorly differentiated carcinoma, or signet ring cell carcinoma. Fifteen cases (68.18 %) contained por/sig components, and the other 7 (31.82 %) contained pap components. Eleven cases showed the G-type, 10 cases showed the GI-type, only one case showed the N-type, and none of them showed the I-type. Among the 10 GI-type cases, 9 cases showed the GI-G type, and one showed the GI-I type. Almost all the 22 patients showed the G- and GI-G types, which was significantly higher than the number of I-type patients (*P* = 0.011). In addition, the 22 patients had a higher proportion of infiltration into the submucosa (*P <* 0.001). (Table 3; Figs. 3 and 4).

**Table 3:** Relationship between Biological Behavior and Mucin Phenotypes

	tub	tub-por/sig	pap/tub-pap	χ2 value	*P* value
**Depth of Invasion**				15.824	0.000
M	219(93.2 %)	9(60.0 %)	5(71.4 %)		
SM	16(6.8 %)	6(40.0 %)	2(28.6 %)		
**Mucin Phenotype**				14.743	0.011
G-type	43(18.3 %)	7(46.7 %)	4 (57.1 %)		
GI-type	134(57.0 %)	7(46.7 %)	3(42.9 %)		
I-type	51(21.7 %)	0(0.0 %)	0(0.0 %)		
N-type	7(3.0 %)	1(6.6 %)	0(0.0 %)		

*M* mucosa, *SM* submucosa

### Follow-up

Six patients underwent additional gastrectomy, and there was no residual tumour or lymph node metastasis. All patients were under close follow-up, and neither recurrence nor metastasis was detected.

## Discussion

The mucin phenotype classification is based on the mucin marker expression profile. After year 2000, the gastric and intestinal mucin phenotypes were analysed by IHC [15]. The mucin markers MUC5AC, MUC6, MUC2 and CD10 were considered necessary, although there is no consensus on the number of markers that should be used to define a mucin phenotype or the percentage of tumour cells that must be stained [6–8, 11, 15, 18]. MUC5AC is a secreted mucin expressed in the surface mucous epithelium of normal gastric mucosa. High expression of MUC6 is observed in fundic mucous neck cells and pyloric glands of gastric mucosa. CD10 is a marker for the brush border on the luminal surface of the small intestine. In the normal adult intestine, MUC2 expression is observed in the perinuclear areas of goblet cells.

We showed that the expression of MUC5AC, MUC6, MUC2 and CD10 was detected in 167 (64.98 %), 187 (72.76 %), 164 (63.81 %), and 112 (43.58 %) of the 257 EGCs, respectively. In previous studies, the expression percentages of MUC5AC, MUC6, MUC2 and CD10 in GCs were 55.1-67.5 %, 44.9-64 %, 35.4-49.3 % and 20.6-20.9 %, respectively [19, 20], and for EGCs, the expression of each mucin marker was 68.75-96.8 %, 19.6-71.58 %, 25-62.10 %, and 0-79 %, respectively [6, 11, 21].

Based on the combinations of expression of these markers, the 257 EGCs were classified into the G-type (21 %, 54/257), GI-type (56 %, 144/257), I-type (20 %, 51/257) and N-type (3 %, 8/257); in previous reports, the incidence percentages of each of these mucin phenotypes were found to be 15-41.1 %, 20.3-60.1 %, 18.5-46.6 %, and 3.7-31.6 %, respectively, in advanced GCs [3, 13, 14], and 7.9-36.8 %, 18.8-41.2 %, 15.4-55.56 %, and 0-11.1 %, respectively, in early- stage GCs [7, 11, 19–25]. Our results were consistent with these studies. The reported expression ranges vary greatly among different investigators, and different markers, antibodies and case groups may account for this discrepancy. Koyama et al. reported that the incidence of G-type was 19.3 % [26], which was similar to that found in the present study (21 %); however, in his report, the incidence of I-type was much higher than that of G-type (43.8 % vs. 19.3 %), as was reported by Fabio et al. [11]. While Tajima et al. [22] reported the opposite result, since in their study, the incidence of the G-type was much higher than that of the I-type (36.8 % vs. 15.4 %). In our study, the incidence of G-type was almost the same as that of I-type (21 % vs. 20 %). Overall, based on our data, much more than half of these cases were classified as G- and GI-G type GC (61.09 %, 157/257), which is much higher than the incidence of I- and GI-I type GC (36.96 %,95/257). A previous report revealed that almost all intramucosal GC cases exhibited the gastric phenotype, including the GI phenotype [15].

The relationship between mucin phenotypes and clinicopathological features was investigated. We found that histology classification (both the JGCA and WHO classification) was closely related to the mucin phenotype. The incidence of I-type was greater than those of the G-, GI- and N-type (100.0 % vs. 79.7 %, 93.1 %, 87.5 %) in differentiated tubular adenocarcinoma. The G-type was histologically significantly correlated with the mixed type (with poorly differentiated/papillary carcinoma). Our data showed that the proportion of G-type carcinoma increased during the transition from solely differentiated type to mixed type carcinoma. Mixed-type early-stage carcinoma more frequently expressed G-type mucin, and G-type tumours were associated with a higher rate of undifferentiated-type tumours than I-type tumours [7, 22].

There were no significant differences between mucin phenotypes and other parameters, including sex, age, margin, colour, tumour size, gross type, depth of invasion, and lymphovascular invasion (*P* > 0.05). These results are consistent with those of other studies in the literatures [22, 24, 27], and there was no clear correlation between phenotypes and clinicopathological characteristics, including sex, age, tumour size, location, macroscopic features, lymphatic or venous invasion, or lymph node metastasis in the case of the differentiated type [22, 24, 27]. Koseki et al. [7] and Oya et al. [28] reported that the incidence of lymphatic invasion, venous invasion and lymph node metastasis in gastric phenotype carcinomas was significantly higher than that in intestinal phenotype carcinomas. In addition, G-type EGCs were correlated with some distinct macroscopic features, namely, a smaller tumour diameter [15], discoloured surface and non-wavy tumour margins [23, 29]. G-type differentiated adenocarcinomas showed a depressed type, indistinct margins and monotonous colour tone across the mucosal layer, whereas I-type adenocarcinomas had an elevated, distinct margin and a red mucosa [3, 4, 30]. The discrepancy of these results may have been due to heterogeneous components that contained poorly differentiated adenocarcinoma [27].

Intestinal metaplasia has been frequently observed surrounding GC, especially differentiated adenocarcinomas. IM has malignant potential and has been regarded as a precursor of gastric neoplasms. According to Laurén, intestinal-type adenocarcinoma is preceded by metaplastic changes, while diffuse-type adenocarcinoma arises in non-IM gastric mucosa [2]. In the current study, background mucosal IM was observed in 79.9 % of cases among the G-, GI- and I-type EGCs and 87.0 % of cases among the G-type EGCs. 25 % of I-type cases arose from the normal mucosa without IM. IM did not significantly differ among mucin phenotypes (*P >* 0.05). However, incomplete and complete IM significantly differed with respect to mucin phenotypes *(P* = 0.004, *P* = 0,018). A total of 77.78 % (42/54) of G-type and 70.6 % (36/51) of I-type patients had complete IM, which was higher than the rates among GI-type patients (83/114, 57.6 %). The expression of incomplete IM in GI-type EGCs was higher than in G- and I-type EGCs (21.5 % vs. 9.3 % vs. 3.9 %). Our results demonstrated a remarkable difference between mucin phenotypes and the background mucosa. Similar results have been reported by Kabashima et al. [31] and Matsuoka [23]. The mucin phenotype of the carcinoma was independent of mucin phenotypic changes in the surrounding mucosa, and the carcinoma may undergo individual intestinalization. The G-type may imitate the surrounding mucosa, and the carcinomas and the background mucosa have an unstable status, as they commonly possess the hybrid phenotype of the stomach and the small intestine [23, 31].

Mucin phenotypes can indicate biological behaviour in GCs. G-type GCs have increased potential for invasion and metastasis due to infiltrating of deeper layers or more surrounding structures, a higher rate of lymph node metastasis, and poorer prognosis [3, 12, 18, 21]. Even differentiated adenocarcinomas of the G-type had similar outcomes, focused on prognoses, as undifferentiated adenocarcinomas [7–10]. In our research, six patients underwent additional gastrectomy, and there was no residual tumour or lymph node metastasis. All patients were under close follow-up, and neither recurrence nor metastasis was detected. The mixed type (mixed with poorly differentiated or papillary adenocarcinoma) was mainly of the G-type, which was significantly higher than that of purely differentiated tubular adenocarcinoma (*P* < 0.05), and the depth of infiltration was deeper (*P* < 0.05). The G-type group had the highest proportion (11/54, 20.37 %) with poorly differentiated/undifferentiated components, and almost all of them (19/22, 86.36 %) expressed the G- and GI-G types. The mixed type may represent a progressive loss of glandular structure during progression of the cancer from the mucosa to advanced stage, and those with submucosal invasion was a risk factor for lymph node metastasis [7, 22, 32]. Differentiated EGC of G-type frequently changed histologically into signet ring-cell carcinoma or poorly differentiated adenocarcinoma. These results may imply more aggressive biological behaviour and poorer prognosis.

## Conclusions

Our study reports the expression of mucin markers (MUC5AC, MUC6, MUC2 and CD10) and mucin phenotypes in differentiated EGC samples from ESD/EMR in the Chinese population. Mucin phenotypes of early differentiated gastric cancer are of clinical significance, and G-type GC exhibits aggressive biological behaviour in early differentiated GCs, especially in those with poorly differentiated adenocarcinoma or papillary adenocarcinoma components.

## Data Availability

The data sets used and/or analyzed during the current study are available from the corresponding author on reasonable request.

## Abbreviations

GC: Gastric cancer
EGC: Early gastric cancer
WHO: World Health Organization
JGCA: Japanese Gastric Cancer Associatio
EMR: Endoscopic mucosal resection,
ESD: Endoscopic submucosal dissection
G-type: Gastric-phenotype
GI-type: Gastrointestinal-phenotype
I-type: Intestinal-phenotype
N-type: Null-type
GI-G type: Gastric-predominant GI phenotype,
GI-I type: Intestinal-predominant phenotype
IM: Intestinal metaplasia
pap: Papillary adenocarcinoma
tub: Tubular adenocarcinoma
well-diff/tub 1: Well differentiated tubular adenocarcinoma
mod-diff/tub 2: Moderately differentiated tubular adenocarcinoma
por: Poorly differentiated
sig: Signet-ring cell
muc: Mucinous
